# Draft Genome Sequence of the Oyster Larval Probiotic Bacterium *Vibrio* sp. Strain OY15

**DOI:** 10.1128/genomeA.01006-14

**Published:** 2014-10-02

**Authors:** Harold J. Schreier, Eric J. Schott

**Affiliations:** a Department of Marine Biotechnology, Institute of Marine and Environmental Technology, University of Maryland Baltimore County, Baltimore, Maryland, USA; b Department of Biological Sciences, University of Maryland Baltimore County, Baltimore, Maryland, USA; c University of Maryland Center for Environmental Science, Institute of Marine and Environmental Technology, Baltimore, Maryland, USA

## Abstract

We report the draft genome sequence of *Vibrio* sp. strain OY15, a Gram-negative marine bacterium isolated from an oyster (*Crassostrea virginica*) digestive tract and shown to possess probiotic activity. The availability of this genome sequence will facilitate the study of the mechanisms of probiotic activity as well as virulence capacity.

## GENOME ANNOUNCEMENT

Controlling microbial pathogens in aquaculture using probiotic bacteria is becoming increasingly preferred over the use of chemical treatments, such as disinfectants or antibiotics (1). The marine *Vibrio* sp. OY15 is a naturally occurring bacterium isolated from the digestive gland of an adult oyster (*Crassostrea virginica*) and has been shown to significantly improve survival of oyster larvae to metamorphosis when challenged with a pathogenic *Vibrio* strain (2). Here we announce the genome sequence of strain OY15 in order to facilitate identification of processes involved in probiotic activity and to ascertain virulence potential.

A single colony of strain OY15 was grown in marine broth 2216 (Difco) at 28°C and DNA was extracted using the Wizard genomic DNA purification kit (Promega). Sequencing was done using an Illumina MiSeq benchtop sequencer. The read library contained 9,221,838 paired-end reads with 284 average read length and average coverage of 430×. *De novo* assembly of the paired reads was done using CLC Genomics Workbench (CLC Bio/Qiagen) yielding 45 contigs with an average length of 115,533 bp and 5,198,998 total bp. The *N*_50_ is 544,789 bp with a G+C composition of 44.6%. Gene prediction and annotation using RAST (Rapid Annotation using Subsystem Technology) (3), generated 4,814 open reading frames. The closest neighbors identified by SEED viewer 2.0 (4) were *Vibrio* sp. EX25 (score = 534) and *V. alginolyticus* 40B (score = 489).

The genome carries genes for regulators *luxU*, *luxO*, and *hapR* and a homoserine lactone efflux pump—suggesting that quorum sensing is likely important for colonization (5)—as well as *scrABC* systems involved in *Vibrio* swarming (6). A ferric siderophore transporter, vibrioferrin/ferrichrome siderophores, and the Fur and IrgB regulators may also play roles in colonization of the digestive tract (7). A cluster of 17 genes for mannose-sensitive hemaglutinin (MSHA) biogenesis proteins were identified, which may also play roles in adhesion and colonization (8). Candidates for probiotic activity include several Rhs family genes, which mediate intercellular competition (9) and may stimulate host immunity (10), and three alginate lyase precursors, which disrupt biofilms (11). Gene clusters associated with exopolysaccharide production—*rbm* and *vps*—are present, which may play a role in antibiofilm activity (12). Bacteriocin (colicin V) and bacteriocin tolerance genes were also found.

While phage-related genes encoding putative RTX and zona occludens toxins were identified in the OY15 genome, genes encoding *Vibrio* CTX phage (13) appear to be absent. Virulence-related secretory HlyD, several hemolysins, the *toxRS* and *vieSB* virulence regulators, and genes encoding types I, III, and VI secretion system components were found. On the other hand, *tdh* and *trh* genes associated with virulent *V. parahaemolyticus* (14, 15) were not identified, which is consistent with the finding that OY15 tested negative in a mammalian-cell bioassay for cytotoxity (J. Jones, U.S. FDA, personal communication). Genomic and phenotypic studies of OY15 will improve our understanding of probiosis and pathogenesis mechanisms of *Vibrio* spp.

### Nucleotide sequence accession numbers.

This whole-genome shotgun project has been deposited at DDBJ/EMBL/GenBank under accession number JPIL00000000. The version described in this paper is the first version, JPIL01000000.
